# Optimizing treatment administration strategies using negative mNGS results in corticosteroid-sensitive diffuse parenchymal lung diseases

**DOI:** 10.1016/j.isci.2024.110218

**Published:** 2024-06-07

**Authors:** Chuwei Jing, Yuchen Ding, Ji Zhou, Qun Zhang, Mingyue Wang, Qiuxiang Ou, Jia Liu, Ting Xv, Chunlai Feng, Dongmei Yuan, Ting Wu, Ting Weng, Xiaoyong Xv, Shanlin Dai, Qian Qian, Wenkui Sun

**Affiliations:** 1 Department of Respiratory Medicine, Jiangsu Province Hospital/Nanjing Medical University First Affiliated Hospital, Nanjing, Jiangsu, China; 2 Jiangsu Health Vocational College, Nanjing, Jiangsu, China; 3 Research & Development, Dinfectome Inc., Nanjing, Jiangsu, China; 4 Department of Respiratory Medicine, School of Southeast University Affiliated Nanjing Chest Hospital, Nanjing, Jiangsu, China; 5 Department of Respiratory and Critical Care Medicine, Third Affiliated Hospital of Soochow University, Changzhou, Jiangsu, China; 6 Department of Respiratory Medicine, Jinling Hospital, Nanjing University School of Medicine, Nanjing, China; 7 Department of Respiratory Medicine, Zhongda Hospital, School of Medicine, Southeast University, Nanjing, Jiangsu, China; 8 Nanjing Drum Tower Hospital Group Suqian Hospital, Jiangsu, China; 9 Second Affiliated Hospital of Nanjing University of Chinese Medicine, Jiangsu, China

**Keywords:** Medical microbiology, Pharmacology, Cancer

## Abstract

Timely adjustments of antibiotic and corticosteroid treatments are vital for patients with diffuse parenchymal lung diseases (DPLDs). In this study, 41 DPLD patients with negative metagenomic next-generation sequencing (mNGS) results who were responsive to corticosteroids were enrolled. Among these patients, about 26.8% suffered from drug-induced DPLD, while 9.8% presented autoimmune-related DPLD. Following the report of the negative mNGS results, in 34 patients with complete antibiotics administration profiles, 79.4% (27/34) patients discontinued antibiotics after receiving negative mNGS results. Moreover, 70.7% (29/41) patients began or increased the administration of corticosteroid upon receipt of negative mNGS results. In the microbiota analysis, *Staphylococcus* and *Stenotrophomonas* showed higher detection rates in patients with oxygenation index (OI) below 300, while *Escherichia* and *Stenotrophomonas* had higher abundance in patients with pleural effusion. In summary, our findings demonstrated the clinical significance of mNGS in assisting the antibiotic and corticosteroid treatment adjustments in corticosteroid-responsive DPLD. Lung microbiota may imply the severity of the disease.

## Highlights

• Negative mNGS results can effectively confirm patients’ non-infected status
• Negative mNGS results can prompt timely corticosteroid administration and adjustment
• Lung microbiota may imply the severity of DPLD patients

## Graphical abstract

**Figure:**
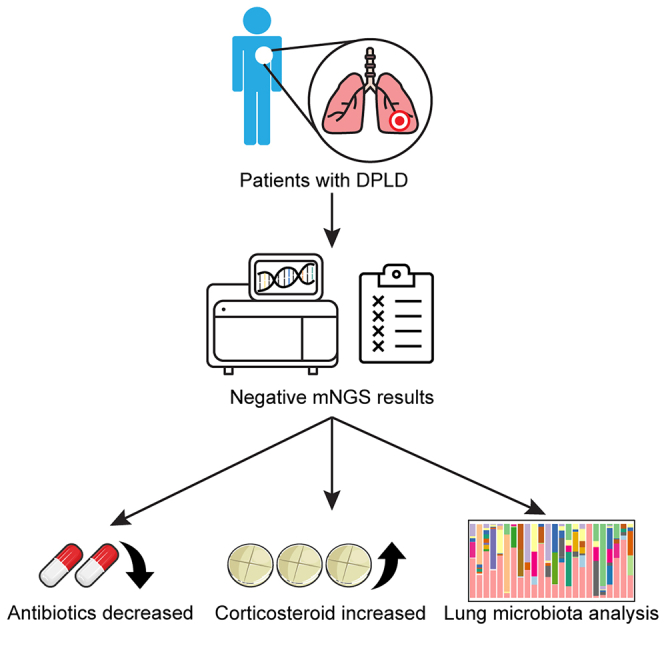

## Introduction

Antibiotic overuse has remained a persistent concern in clinical practice. Notably, antimicrobials are prescribed to half of hospitalized patients.^1^ Up to half of patients hospitalized for infections receive unnecessary, excessive, or suboptimal antibiotics.^2^^,^^3^ In addition to fostering antibiotic resistance, the overuse of antibiotics appears to be associated with elevated mortality rate and other poor outcomes, including longer hospital stay, greater cost, and increased risk of Clostridioides difficile infection (CDI).^4^ Moreover, prolonged antibiotic therapy is associated with an increased risk of adverse events, with each additional day of treatment carrying a 4% higher probability of such events.^5^

Diffuse parenchymal lung diseases (DPLDs), also known as interstitial lung disease (ILD), encompass more than 200 disorders chiefly affecting the pulmonary interstitium.^6^ DPLD could stem from exposure-related, autoimmune-related, drug-related, and other reasons.^7^ Infection serves as a significant etiological factor for DPLD. The clinical presentations and radiological features of both infectious and noninfectious forms of DPLD exhibit remarkable similarities.^8^ Therefore, in clinical practice, there is a tendency to empirically prescribe antimicrobial agents before definitively ruling out the possibility of infection in DPLD patients. However, corticosteroids are the most commonly administered therapy,^8^ which could be used for preemptive treatment of DPLD to relieve symptoms. In terms of the medical property of corticosteroids, it is essential to ensure that patients are in a noninfected state or minimize the use of antibiotics whenever possible before initiating corticosteroid therapy.^9^

Metagenomic next-generation sequencing (mNGS) has been emerged as a powerful tool applied to diagnose infectious diseases in recent years,^10^ which exhibits a higher sensitivity compared to traditional detection methods.^11^^,^^12^ Prior studies have shown that mNGS could facilitate medication modification.^13^ In clinical practice, instances of negative mNGS results may arise, indicative of the absence of clinically significant pathogens. Medical modification could be dependent on these results.^14^ Moreover, compared to 16S rRNA, mNGS furnishes a broader spectrum of information about the background microbiota,^15^ which may be associated with the manifestations of the disease.

In this study, we retrospectively studied the clinical information of corticosteroid-responsive DPLD patients who had negative results of BALF mNGS testing. We aimed to explore the clinical utility of negative mNGS results in medication modification in DPLD and to assess the significance of the identified microbiota.

## Results

### Patient characteristics

**Figure 1:**
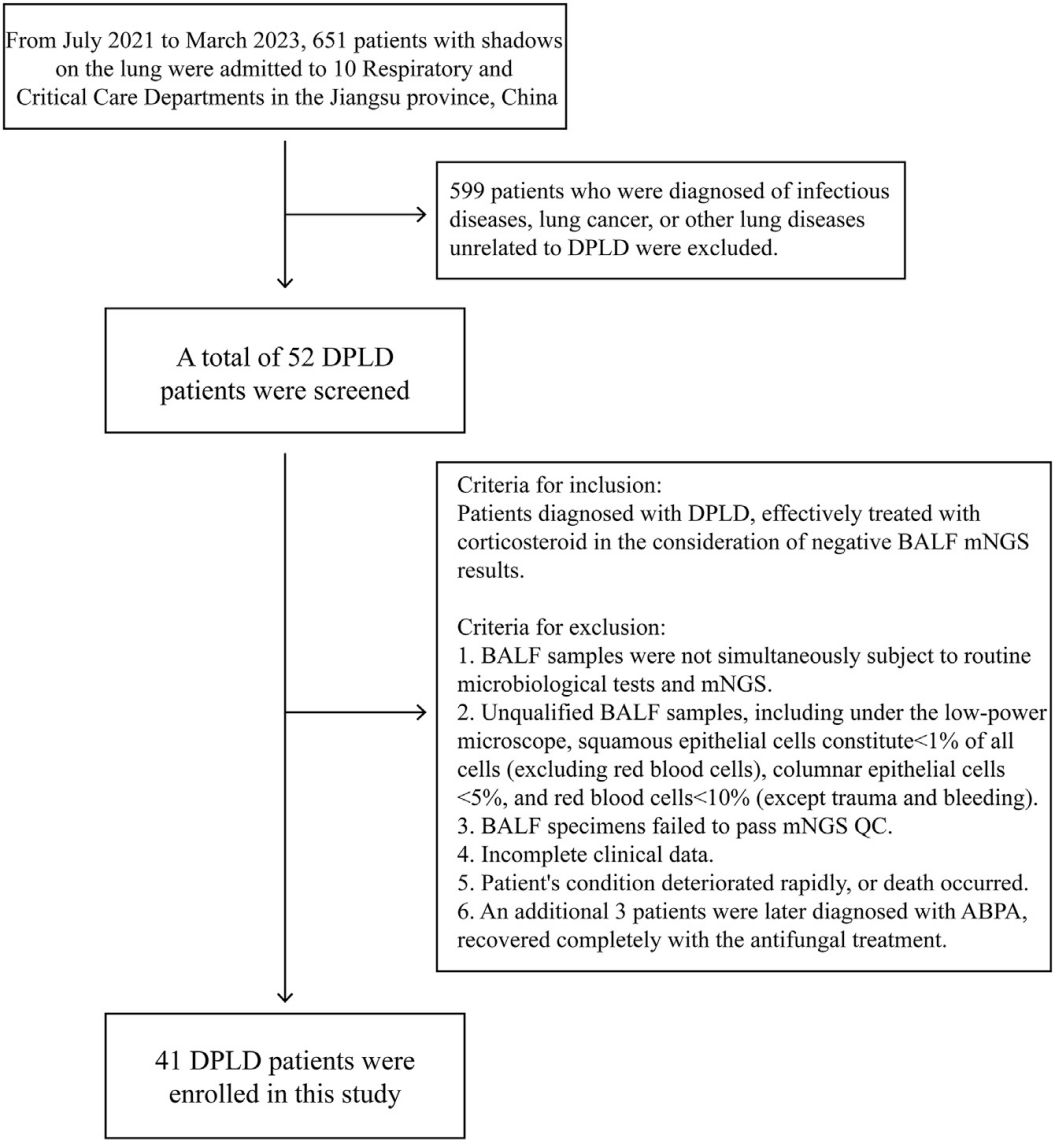
Patient enrollment

During the study, a total of 41 subjects diagnosed with DPLD were identified, all of whom tested negative results for BALF mNGS. (Figure 1; Table 1). The mean age was 65.97 (58–76) years; 60.98% patients (25/41) were males and 56.09% patients (23/41) had comorbidities, including tumor, dermatomyositis, and other diseases. 12 patients had a history of DPLD-related medication, containing methotrexate (MTX), epidermal growth factor receptor (EGFR) inhibitors, and PD1 inhibitors. All the participants received corticosteroids and antibiotics treatment. The median APACHE II score was 12.23 (9–16.5). Elevated C-reactive protein (CRP) was observed in 68.29% (28/41) of patients and only 2 patients were identified with elevated procalcitonin (PCT) (4.9%).

**Table 1:** Characteristics of 41 DPLD patients with negative BALF mNGS results

Characteristics	Patients (*n* = 41)
**Age (years)**	65.97 (58–76)

**Biological sex**	

Male	25 (60.98%)
Female	16 (39.02%)

**Comorbidities**	

Tumor	7 (17.07%)
Dermatomyositis	2 (4.88%)
Rheumatoid arthritis	2 (4.88%)
Diabetes	4 (9.76%)
Other diseases	14 (34.15%)
No	18 (43.91%)

**History use of drugs associated with DPLD**	

MTX	1 (2.43%)
EGFR inhibitor	6 (14.63%)
PD1 inhibitor	5 (12.20%)
No	31 (75.61%)

**Clinical manifestations**	

Fever	11 (26.83%)
Dyspnea	7 (17.07%)
Cough	36 (87.81%)
Expectoration	28 (68.29%)
Breathlessness	22 (53.66%)
Chest distress	24 (58.54%)

**Inflammation biomarker**	

WBC (ˆ10^9^/L)	7.91 (4.44–9.47)
CRP (mg/L)	69.35 (16.38–120.75)
PCT (ug/L)	0.22(0.10–0.23)
**OI (mmHg)**	216.17 (157.14–270.61)

**Corticosteroid therapy**	

Total dosage (mg)	408 (240–758)
Dosage before NGS (mg)	40 (0–160)
Dosage after NGS (mg)	410 (185–640)
Maximum dosage/d (mg)	74.39 (40–80)
Course, Days	10.51 (6–13.5)
**APACHE II score**	12.23 (9–16.5)

**Radiological features on chest CT**	

Ground-glass opacity	19 (46.34%)
Interstitial pattern	17 (41.46%)
Nodules	7 (17.07%)
Pleural effusion	16 (39.02%)
Pleural thickening	18 (43.9%)

**Conventional microbal tests**	

Negative	41(100%)
Positive	0

**Length of stay (days)**	

In ICU	11.47 (7.5–14)
In hospital	16.44 (11–22)

The prescribed dosages of corticosteroids were converted into equivalent methylprednisolone.

OI: SpO2/FiO2.

**Figure 2:**
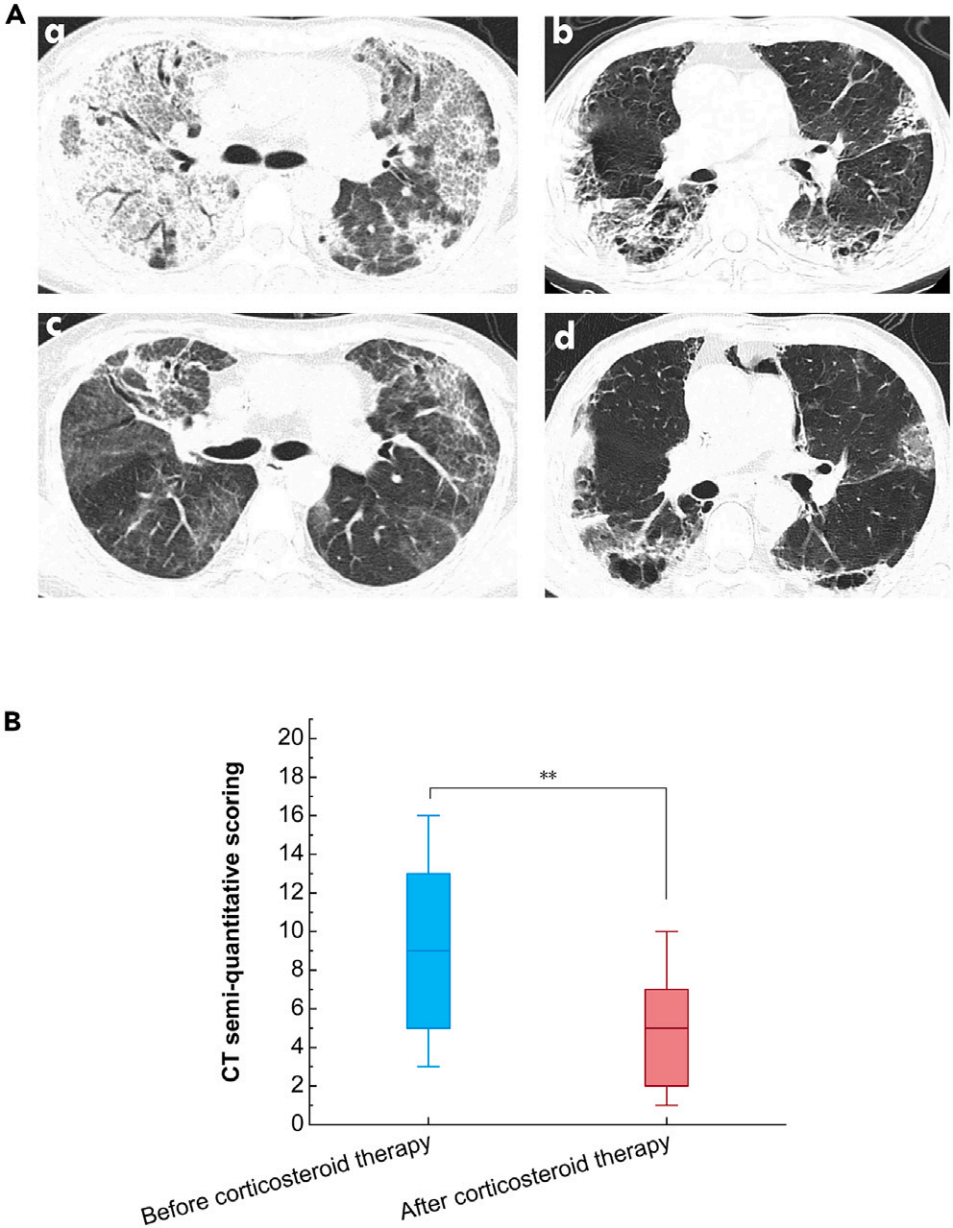
Chest CT images of two representative cases (A) 33-year-old female patient and a 76-year-old male patient were admitted to the RICU with a chief complaint of cough, sputum, and chest tightness. Chest CT showed diffuse parenchymal lung diseases. We performed a bronchoscopy shortly after admission and submitted BALF specimen for metagenomic next-generation sequencing (mNGS) analysis. Based on their negative mNGS results and clinical data, they were administrated with systemic corticosteroid intensive therapy. (A and C) The female patient’s chest CT before and after the report of the negative mNGS result. (B and D) The male patient’s chest CT before and after the report of the negative mNGS result. (B) The semi-quantitative CT scores of 41 patients exhibited a significant decrease after corticosteroid treatment. Note: ∗: *p* < 0.05; ∗∗: *p* < 0.01; ∗∗∗: *p* < 0.001.

Chest CT data demonstrated that ground-glass opacity (46.34%) and interstitial patterns (41.46%) were mostly frequently seen in this cohort with two representative images shown in Figure 2A. Furthermore, CT semi-quantitative scores after corticosteroid therapy were much lower than before the therapy (Figure 2B). In addition, those patients showed elevated CRP (median, 69.35 mg/L), and the median oxygenation index (OI) was 216.17 (157.14–270.61) mmHg (Table 1).

### Negative mNGS results optimized antibiotic therapy strategy adjustment

**Figure 3:**
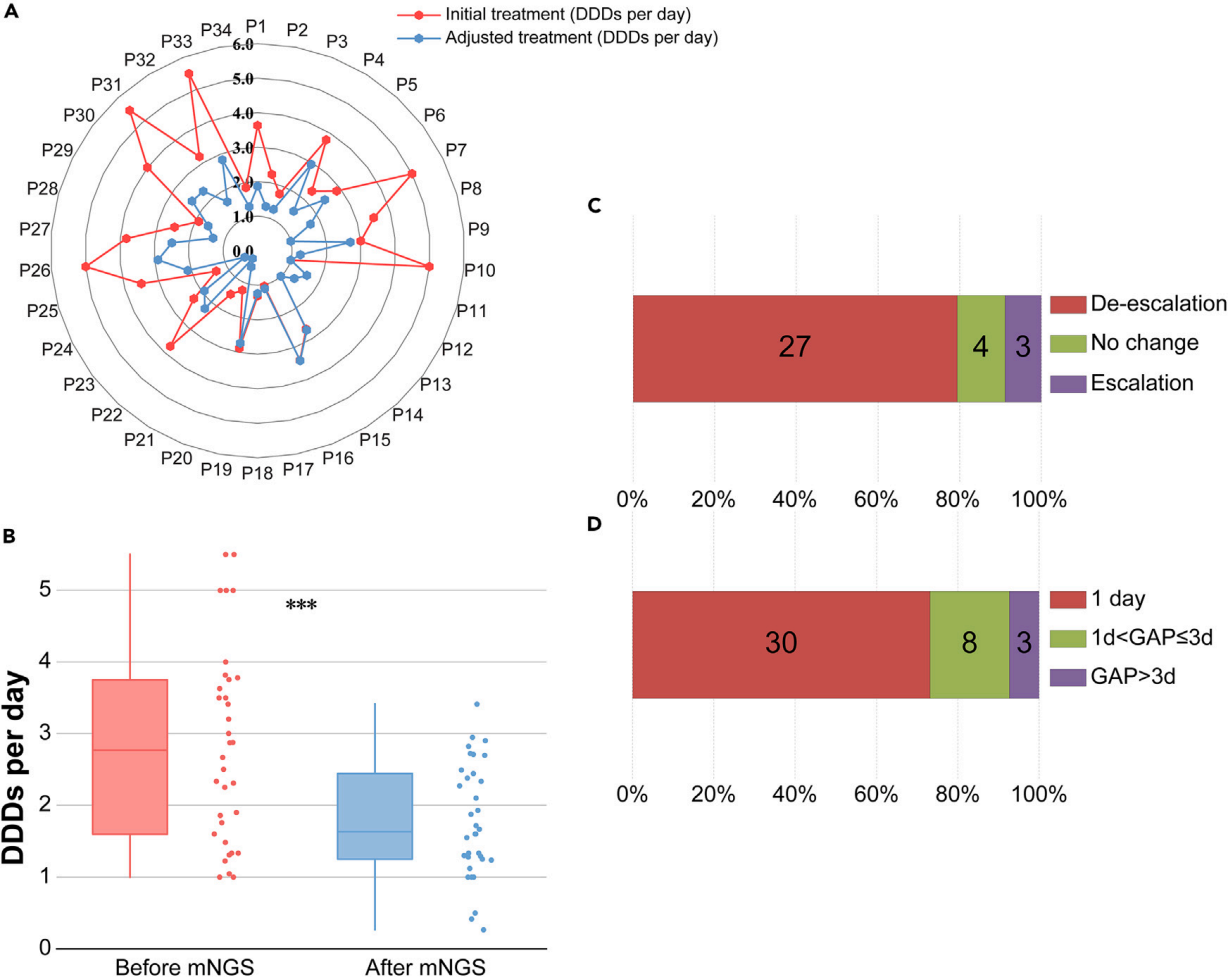
Adjustment of antimicrobial treatments (A) The defined daily doses (DDDs) per day were applied to demonstrate daily antibiotic use for each patient. (B) DDDs significantly declined after the report of the negative mNGS results. (C) The adjustments of the antimicrobial categories. (D) “GAP” was introduced to exhibit the interval time between the receipt of mNGS reports and the subsequent adjustments in antimicrobial treatment. Note: ∗: *p* < 0.05; ∗∗: *p* < 0.01; ∗∗∗: *p* < 0.001.

The alterations of antimicrobial interventions were illustrated in Figure 3. We applied defined daily doses (DDDs) to show the daily use of antibiotics. 7 patients were transferred to our hospital from other medical facilities for treatment, and their previous antibiotic information was unavailable, making it challenging to calculate their corresponding DDDs. Among the remaining 34 patients, 79.4% patients (27/34) downgraded antibiotics, 11.8% patients (4/34) did not change antibiotics, and 8.8% patients (3/34) upgraded antibiotics (Figure 3A). The DDDs following mNGS reports were significantly lower than before (*p* < 0.0001, Figure 3B). The term “GAP” was introduced to show the interval time between the receipt of mNGS reports and the subsequent adjustments in antibiotic treatment. The categories of antibiotics and GAP were presented in Figures 3C and 3D. It is observed that 73.17% patients (30/41) had a GAP of 1 day, 19.51% patients (8/41) experienced a GAP of more than 3 days, and the remaining patients exhibited a GAP ranging from 1 to 3 days (Figure 3D).

### Negative mNGS results adjunct corticosteroid medication adjustment

**Figure 4:**
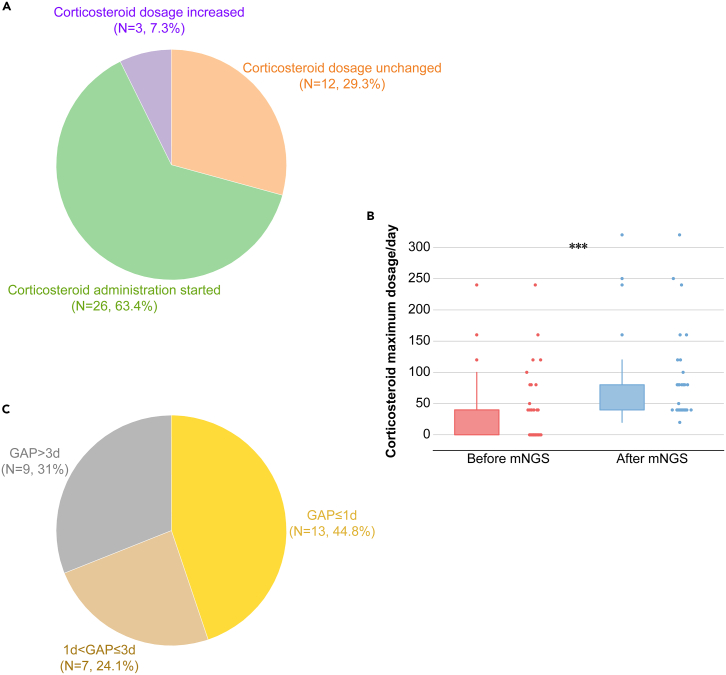
Adjustment of corticosteroid medication (A) Corticosteroid dosage adjustments of the patients. (B) Corticosteroid dosage significantly decreased after the report of the negative mNGS results. (C) “GAP” referred to the interval time between the between when the receipt of mNGS reports and the subsequent adjustments in corticosteroid dosage. Note: ∗: *p* < 0.05; ∗∗: *p* < 0.01; ∗∗∗: *p* < 0.001.

The adjustment of corticosteroid medication was depicted in Figure 4. Following the report of the negative mNGS results, 63.4% patients (26/41) began the administration of corticosteroid, 7.3% patients (3/41) increased the dosage, and 29.3% patients (12/41) maintained the existing dosage (Figure 4A). There was a significant increase in corticosteroid dosage following the report of the negative mNGS results (*p* < 0.0001, Figure 4B). Among those patients who began corticosteroid administration or increased the dosage, 44.8% patients (13/29) made the adjustments within 1 day, 24.1% patients (7/29) adjusted the dosage in 1–3 days, and 31% patients (9/29) implemented dosage changes exceeding 3 days (Figure 4C).

### Negative mNGS results aided in adjusting medication for patients with varying disease severity

**Figure 5:**
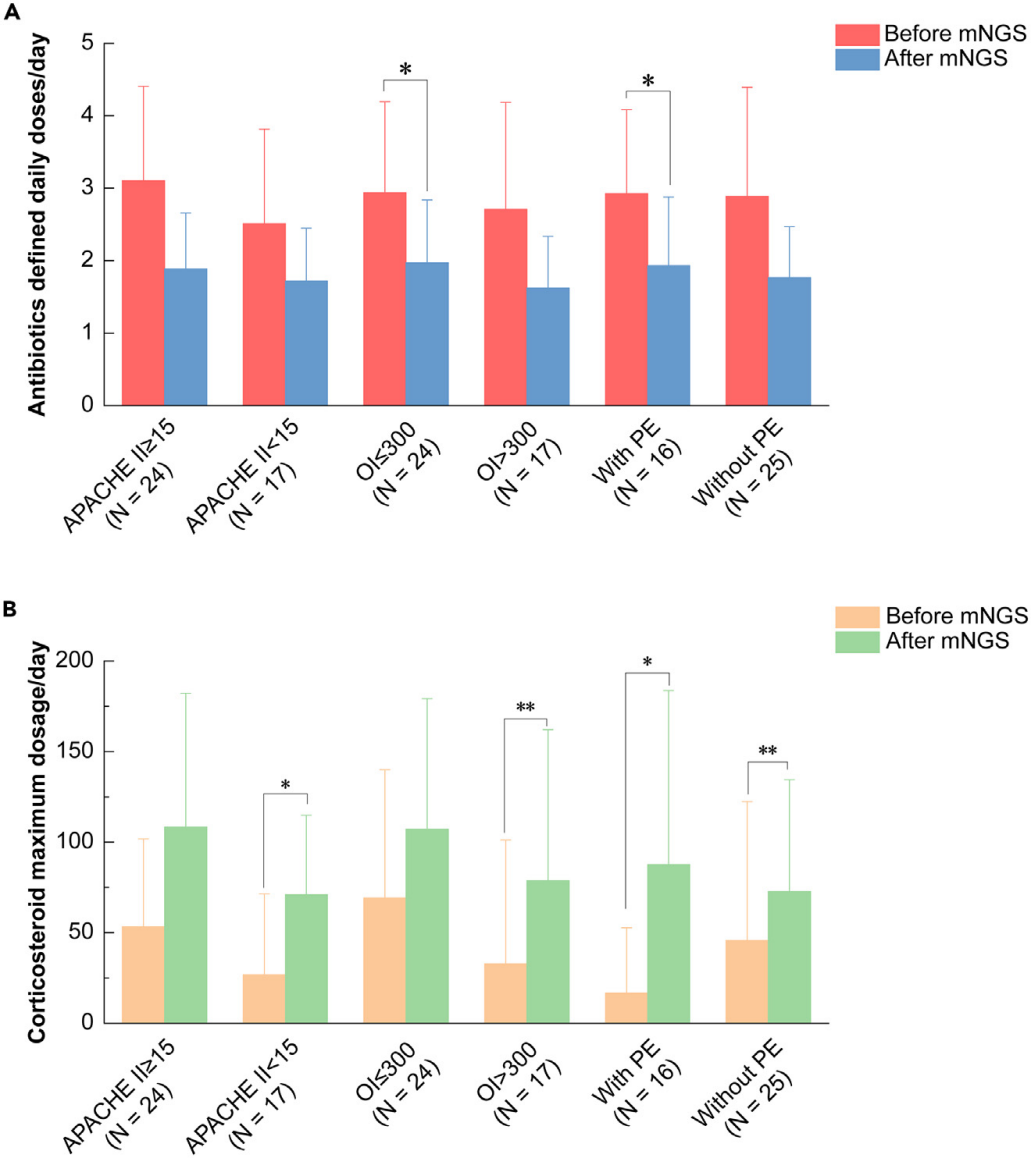
Medication adjustments in patients with varying disease severity before and after negative mNGS results (A) Modification of DDDs per day. (B) Adjustment of corticosteroid maximum dosage/d. Note: APACHE II ≥ 15, *n* = 24, 58.54%; APACHE II < 15, *n* = 17, 41.46%; OI ≤ 300, *n* = 24, 58.54%; OI > 300, *n* = 17, 41.46%; With PE, *n* = 16, 39.02%; Without PE, *n* = 25, 60.98%; Note: OI: oxygenation index, SpO2/FiO2. PE: pleural effusion. Note: ∗: *p* < 0.05; ∗∗: *p* < 0.01; ∗∗∗: *p* < 0.001.

The impact of negative mNGS results on medication adjustments in corticosteroid-responsive DPLD patients motivated us to explore its potential in supporting medication adjustments in both mild and severe cases. To this end, we categorized patients by disease severity and compared changes in DDDs and corticosteroid maximum dosage/day before and after receiving negative mNGS reports. As illustrated in Figure 5, negative mNGS results could effectively adjust the medication in corticosteroid-responsive DPLD patients with varying disease severity.

### Microbiota analysis by mNGS in corticosteroid-responsive DPLD

We analyzed the microbial composition and microbiome differences in the BALF of corticosteroid-responsive DPLD patients. Furthermore, we explored the potential correlation between the detected microbiota and the clinical manifestations of the patients. Our results revealed distinct microbiota between individuals with OI above and below 300, as well as those with and without pleural effusion.

The 5 predominant species by relative abundance were *Lautropia mirabilis*, *Streptococcus parasanguinis*, *Pseudomonas aeruginosa*, *Rothia mucilaginosa*, and *Veillonella parvula* (Figure S1). The microbial species identified were depicted in Figure S2A. α-Diversity and β-diversity were employed to analyze the differences in species diversity between two groups. There was no significant difference in β-diversity (*p* > 0.05). ACE (*p* = 0.014, Figure S2B) and Chao1 (*p* = 0.017, Figure S2C) indexes exhibited an evident difference, suggesting a noteworthy distinction in species composition between the groups, highlighting the necessity of the grouping. The distribution of species was shown in Figure S3A. On the species level, we found *Pseudomonas aeruginosa* was abundant in patients with OI below 300, while *Streptococcus pneumoniae* had higher abundance in patients with OI above 300 (Figure S3B). On the genus level, we detected a pronounced enrichment of *Pseudomonas*, *Corynebacterium*, *Staphylococcus*, and *Stenotrophomonas* among patients with OI below 300, while *Porphyromonas* was enriched in patients with OI above 300 (Figure S3C).

Analysis of microbiome differences between patients with and without pleural effusion was illustrated in Figures S4 and S5. The microbial species distinguished at the species were shown in Figure S4A. There was no significant difference in β-diversity (*p* > 0.05). ACE (*p* = 0.023, Figure S4B) and Chao1 (*p* = 0.014, Figure S4C) indexes demonstrated a noticeable disparity, indicating a significant variation in species composition between the groups. We analyzed species differences between patients with and without pleural effusion (Figure S5A). The distinct microbial profiles at species and genus levels between the two groups were presented in Figures S5B and S5C. At the genus species level, we found *Escherichia* and *Stenotrophomonas* in higher relative abundance in patients with pleural effusion, while *Streptococcus* in higher abundance in patients without pleural effusion.

## Discussion

DPLDs comprise various diseases characterized by diffused lesions of pulmonary parenchyma as visualized on chest high-resolution computed tomography (HRCT). These conditions predominantly target pulmonary interstitium and alveolar cavity.^16^ Among the patients enrolled in this study, 26.8% of patients had a history of use of MTX, PD-1inhibitor, and EGFR inhibitor, which may lead to drug-induced DPLD.^17^ About 9.8% of patients suffered from rheumatoid arthritis or dermatomyositis, which could trigger autoimmune-related DPLD.^8^

Mostly, infection is a primary factor contributing to DPLD. During the treatment of infection, the use of corticosteroids is not recommended as it may aggravate the condition.^18^^,^^19^^,^^20^ However, corticosteroids are the most frequently administrated treatment for DPLD.^8^ Ensuring that DPLD patients are in a non-infected state is essential prior to subsequent corticosteroids therapy. In addition, early intervention plays a vital role as DPLD may bring about an accelerated progressive decline in lung function, respiratory failure and even death.^21^ Previous studies have displayed the efficacy of mNGS in assisting in confirming low probability of infection and guiding antimicrobial de-escalation and discontinuation in patients with rheumatic diseases^13^ and critically ill patients.^14^ Yan et al. discovered that incorporating mNGS tests into conventional microbiologic methods was related to lower rates of antibiotic escalation and may potentially aid in cessation of empirical antiviral therapies in lower respiratory tract infections (LRTIs) patients.^22^ Meanwhile, mNGS could benefit the patients after allogeneic hematopoietic stem cell transplantation by lowering the mortality of pulmonary complications on account of its early accurate diagnosis and the ability to discern the pathogens.^23^ In this study, we evaluated the potential application value of mNGS negative results in patients with corticosteroid-responsive DPLD. Specifically, we revealed the utility of mNGS in assisting antibiotic adjustments and its ability to detect lung involvement and differential flora as biomarkers for diagnosis in these patients.

Antimicrobial strategies are essential for critically ill patients. Precise and timely antibiotic treatment is required to avoid unintended adverse effects.^24^ mNGS had a high negative predictive value (NPV) exceeding 95% for viruses in patients with virus pneumonia. This implied that pneumonia could be ruled out after obtaining negative mNGS results.^25^ Zhou et al. revealed that mNGS could guide antibiotic de-escalation in 25.5% of 159 patients with pneumonia in a before-after study.^26^ Our study inaugurally revealed that the mNGS negative results could assist in confirming the probability of infection and further facilitate antimicrobial adjustments in DPLD patients. Antibiotic de-escalation occurred in 79.4% patients and 1-day-GAP occurred in 73.17% patients, suggesting the potential utility of mNGS for antibiotic optimization and timely adaptation. For the patients whose antibiotics showed escalation or no change, this may be attributed to the tendency to the elevated use of antibiotics in critically ill patients.^27^ In summary, mNGS is a promising tool to confirm the non-infected status of patients, which could facilitate their subsequent treatment.

The efficacy of corticosteroids in DPLD varies across its broad spectrum of the disease. DPLD patients diagnosed with rheumatoid arthritis and amyopathic dermatomyositis with anti-MDA5 antibodies may drive benefits from corticosteroid treatment.^8^ But evidence-based treatment recommendations are only available for a few diseases within the spectrum of DPLD. It is time consuming to figure out the underlying cause of the DPLD disease, particularly for critically ill patients. Prompt administration of the appropriate initial therapy is the primary goal of most critical care practitioners. In our study, all the patients enrolled timely commenced corticosteroid administration and increased the dosage following the reports of the negative mNGS results. In our study, some patients maintained their corticosteroid dosage, possibly attributed to physicians preemptively administering corticosteroid medication before receiving the reports, using mNGS as an adjunct diagnostic tool. Alterations of radiological imaging and clinical manifestations before and after corticosteroid treatment indicated the amelioration of the patients’ conditions. Preemptive utility of corticosteroids is not radical, as corticosteroids are the most commonly administered therapy for acute exacerbations, which showed a 90-day mortality rate as high as 50% in DPLD patients.^28^ In our study, mNGS negative results aided to guide corticosteroids administration in the corticosteroid-responsive DPLD patients. But in clinical practice, timely identification of disease deterioration in DPLD patients remains challenging, potentially leading to treatment delays.

Increasingly prevalent sequencing technologies have unveiled the relationship between microbiota and diseases. Zhu et al. revealed that gut *Akkermansia muciniphila* (Akk) contributes to the suppression of tumorigenesis in lung cancer,^29^ offering new insights into lung cancer treatment. mNGS reported all the microorganism detected in the sample, encompassing opportunistic pathogen and resident flora in the respiratory tract.^22^ Yuan et al. utilize mNGS to uncover the relationship between lung microbiota and lung diseases in patients with pneumoconiosis and pulmonary infection, implying that lung microbiota could be biomarkers in lung diseases.^15^ CRP and PCT could exclude the infection probabilities in critical ill patients but the predictive performance was vulnerable to the impact of antibiotic utility.^30^ Compared to CRP or PCT, microbiota would serve as better biomarkers for assessing therapeutic efficacy and monitoring disease progression in lung diseases, as they are less influenced by prior antibiotic exposure.^31^

OI (SpO2/FiO2) was proposed as a predictor in critical respiratory diseases.^32^ Besides, the presence of pleural effusion was associated with increased mortality rates among critically ill patients.^33^ Airway dysbiosis could exacerbate decline in lung function in chronic obstructive pulmonary disease (COPD) patients. Besides, targeting the airway microbiota could help slow the progression of COPD.^34^ In our study, it was noteworthy that high detection rate of *Pseudomonas*, *Corynebacterium*, *Staphylococcus*, and *Stenotrophomonas*, as well as low detection rate of *Porphyromonas* has been confirmed in patients with OI below 300. Interestingly, the relative abundances of *Staphylococcus* and *Stenotrophomonas* were identified higher in patients with OI above 300 and in patients with pleural effusion. Previous studies reported that *Staphylococcus* and *Stenotrophomonas* have been associated with disease exacerbation,^35^^,^^36^ while patients with acute exacerbation diseases often experience varying degrees of decreased OI.^37^^,^^38^ In addition, *Staphylococcus* is associated with the development of pleural effusion,^39^ which is consistent with our study. These microbiotas require special attention as they could be potential biomarkers implying condition deterioration. Moreover, we revealed higher abundance of *Escherichia* and *Stenotrophomonas*, along with lower abundance of *Streptococcus* in patients with pleural effusion as opposed to those without this condition. The alterations in microbiota could be ascribed to pulmonary consolidation caused by pleural effusion, which could potentially indicate the emergence of pleural effusion and alert the physicians to implement therapeutic interventions.

Precise and timely treatment could significantly improve the prognosis of critically ill patients with DPLD. This study demonstrated that negative mNGS results could be valuable in guiding antimicrobial and corticosteroid adjustments. Furthermore, the distinct microbiota detected by mNGS could potentially monitor the conditions deterioration. Therefore, this study carries remarkable clinical implications and furnishes beneficial information for physicians. We introduce a flow-chart illustrating a guiding strategy for clinical management of patients with DPLD (Figure S6). Combined with clinical data, we could discern whether mNGS reports are negative or positive. Next, with the proof of detected microbiota by mNGS, we could assess the severity of the disease. Based on the information provided, physicians could accurately select the appropriate and timely therapy.

### Limitations of the study

In this study, we explored the utility of negative mNGS results in corticosteroid-responsive DPLD patients and lung microbiota of these patients. However, the restricted cohort size, complex background, and varied immune and inflammatory responses of the DPLD cohort limit the validity of the microbiome analysis. The mNGS sequencing depth was comparatively lower than in previous lung microbiota research. A prospective cohort study with a larger sample size is needed to investigate the application of mNGS negative results and lung microbiota of DPLD patients in depth. Despite these limitations, we showed that negative mNGS results could aid in adjusting antibiotic administration. Besides, our findings shed light on the microbial composition of corticosteroid-responsive DPLD patients, disease severity, highlighting the differential microbiota between patients with OI above and below 300, as well as those with and without pleural effusion, which could serve as indicators to evaluate disease severity. In summary, our study underscores the significance of negative mNGS results in adjusting medication and evaluating disease severity in corticosteroid-responsive DPLD patients.

## STAR★Methods

### Key resources table

**Table:** 

REAGENT or RESOURCE	SOURCE	IDENTIFIER
**Biological samples**		

BALF samples	Jiangsu Province Hospital, Nanjing, China	N/A

**Chemicals, peptides, and recombinant proteins**		

UltraPure™ DNase/RNase-Free Distilled Water	Thermo Fisher	Cat# 10977015
ethanol	SIGMA	Cat# E7023
Isopropanol	Sangon Biotech	Cat# A507048
Gibco™ PBS pH7.2	Thermo Fisher	Cat# 20012027

**Critical commercial assays**		

TIANamp Micro DNA Kit	Tiangen Biotech	Cat# 4992287
QIAamp Viral RNA Mini Kit	Qiagen	Cat# 52904
Hieff NGS™ OnePot II DNA Library Prep Kit	Yeasen Biotechnology	Cat# 13321
Hieff NGS™ MaxUp rRNA Depletion Kit	Yeasen Biotechnology	Cat# 12257
Hieff NGS™ Ultima Dual-mode RNA Library Prep Kit	Yeasen Biotechnology	Cat# 12308
Qubit™ dsDNA Quantification Assay Kits	Thermo Fisher	Cat# Q32854
Qubit™ RNA Assay Kits	Thermo Fisher	Cat# Q32855

**Deposited data**		

Metagenomic sequencing data	Genome Sequence Archive	CRA013674

**Software and algorithms**		

R software (v4.3.1)	the R Core Team and the R Foundation for Statistical Computing	https://www.r-project.org/
bcl2fastq2 Conversion Software (v2.20)	Illumina Inc.	https://support.illumina.com/downloads/bcl2fastq-conversion-software-v2-20.html
Trimmomatic (v0.36)	Bolger et al.^41^	https://github.com/timflutre/trimmomatic
bowtie2 (v2.2.6)	Langmead and Salzberg.^42^	http://bowtie-bio.sourceforge.net/bowtie2/index.shtml
Kraken (v2.0.7)	Wood et al.^43^	https://github.com/DerrickWood/kraken2/wiki
Bracken (v2.5.0)	Lu et al.^44^	https://github.com/jenniferlu717/Bracken
Blast (v2.3.0)	Boratyn et al.^45^	http://blast.ncbi.nlm.nih.gov.
SPSS statistics (v25.0)	IBM Corporation	http://www.spss.com.hk/software/statistics/
OriginPro (v2023)	OriginLab Corporation	https://www.originlab.com/origin

**Other**		

DIFSEQ-200 sequencer	Dinfectome	N/A
Qubit™ 4 Fluorometer	Thermo Fisher	Cat# Q33238
C1000 Touch™ Thermal Cycler	Bio-Rad Laboratories, Inc	https://www.bio-rad.com/zh-cn/product/c1000-touch-thermal-cycler?ID=LGTW9415

### Resource availability

#### Lead contact

Further information and requests for resources and reagents should be directed to and will be fulfilled by the lead contact, Dr. Wenkui Sun (sunwenkui@njmu.edu.cn).

#### Materials availability

This study did not generate new unique reagents.

#### Data and code availability

• The sequencing data presented in the study are deposited in the Genome Sequence Archive repository (https://ngdc.cncb.ac.cn/gsa/), accession number (CRA013674).
• This paper does not report original code.
• Any additional information required to reanalyze the data reported in this paper is available from the lead contact upon request.

### Experimental model and study participant details

A total of 651 Chinese patients who had shadows on the lung and were admitted to 10 Respiratory and Critical Care Departments in the Jiangsu province (China) between July 2021 and March 2023 were screened. Among them, 41 patients diagnosed with DPLD and effectively treated with corticosteroid (showing improved respiratory symptoms and a reduction of radiographic lesions by more than 10%^40^) with negative BALF mNGS results were enrolled in this study. Patients’ demographic and clinical data, including the age, biological sex, defined daily doses (DDDs) of antibiotics, and CT images confirmed by two experienced pulmonologists, were retrospectively reviewed. Clinicopathological information from all patients is detailed in Table 1 and the overall workflow for this study is illustrated in Figure 1. This study was approved by the Institutional Review Board and Ethics Committee of the Nanjing Medical University [ID: 2023-SR-014; Date: February 28th, 2023; Title: Clinical utility of metagenomic next-generation sequencing in diffuse parenchymal lung diseases].

### Method details

#### Specimen processing

The collected BALF samples were sent for mNGS testing and conventional microbiological tests (CMTs) concurrently.

The mNGS process includes sample pretreatment, nucleic acid extraction, library construction, sequencing, and data analysis. RNA extraction and sequencing would be performed if an RNA virus infection were suspected.

Selected CMTs were performed considering patients’ clinical manifestations, including: smear, culture, respiratory panel (including Respiratory Syncytial Virus [RSV] IgM antibodies, Adenovirus IgM antibodies, Influenza Virus Type A IgM antibodies, Influenza Virus Type B IgM antibodies, Parainfluenza Virus IgM antibodies, Mycoplasma pneumoniae IgM antibodies, Chlamydia pneumoniae IgM antibodies, Legionella pneumophila IgM antibodies), Gram stain test, Galactomannan test, tuberculin skin test, Tuberculin T-cell test, Tuberculosis DNA Test, Influenza virus antigen.

#### Nucleic acid extraction

Samples of sputum/bronchoalveolar lavage fluid (BALF) were collected from patients according to standard procedures. BALF samples were centrifuged at 10,000 g for 5 minutes and discarded the supernatant. Subsequently, the process of removing host DNA from the remaining precipitates was performed. DNA was extracted by using the TIANamp Micro DNA Kit (Tiangen Biotech, Beijing, China) according to the manufacturer’s protocols. Enzyme-free distilled water in a tube was used as the no-template control (NTC) in parallel with the clinical samples. The quantity and quality of DNA were assessed using Qubit (Thermo Fisher Scientific) and NanoDrop (Thermo Fisher Scientific) instruments, respectively.

The QIAamp Viral RNA Mini Kit (Qiagen) was used to extract RNA from BALF, and the library was constructed after Qubit quantification.

#### Library preparation and sequencing

DNA libraries were prepared using the Hieff NGS C130P2 OnePot II DNA Library Prep Kit for MGI (Yeasen Biotechnology) according to the manufacturer’s protocols. For RNA library preparation, the rRNA was removed from the total RNA using the Hieff NGS MaxUp rRNA Depletion Kit (Yeasen Biotechnology), and the libraries were constructed using Hieff NGS RNA Library Prep Kit (Yeasen Biotechnology). After library preparations, both DNA and RNA libraries were qualified by Agilent 2100, and were later 50 bp single-end sequenced on the MGISEQ-200 sequencer (MGI Tech, China). Enzyme-free distilled water was sequenced as the no-template control (NTC) in parallel with the clinical samples.

#### Bioinformatics analysis

We used an in-house developed bioinformatics pipeline for microorganism identification. Raw sequencing data was splited by bcl2fastq2 (version 2.20). High-quality sequencing data were obtained following the removal of low-quality reads, adapter contaminations, and duplicated and short (read length <36 bp) reads using Trimmomatic^41^ (version 0.36). Human host sequences were excluded by mapping to the human reference genome (hs37d5) using bowtie2^42^ software. The remaining data aligned with the NCBI microorganism genome database for microorganism identification by Kraken^43^ (version 2.0.7), and species abundance was estimated by Bracken^44^ (version 2.5.0). The sequences filtered by the Kraken underwent further alignment using the Blast (v2.3.0),^45^ which helped to recapture a portion of the sequences that might have been incorrectly filtered out, ensuring a more comprehensive dataset. The microorganism genome database contained genomes or scaffolds of bacteria, fungi, viruses, and parasites was downloaded from GenBank (release 238, ftp://ftp.ncbi.nlm.nih.gov/genomes/genbank/). Consequently, the microbial compositions of the samples were obtained.

#### Interpretation and reporting

We used the following criteria for positive results of mNGS: (a) For Mycobacterium, Nocardia and Legionella pneumophila, the result was considered positive if a species detected by mNGS had a species-specific read number ≥ 1. (b) For bacteria (excluding Mycobacterium, Nocardia and Legionella pneumophila), fungi, virus and parasites, the result was considered positive if a species detected by mNGS had at least 3 non-overlapping reads. (c) Pathogens detected in the NTC were excluded unless the detected reads in the clinical samples were ≥ 10-fold of respective reads in the NTC.

Negative BALF mNGS results were defined as that, based on patient’s clinical manifestations, microbes detected are not considered to have practical clinical significance or do not provide clinically meaningful etiological information for guiding therapeutic adjustments.^14^

#### Analytical performance of mNGS assay

The detection limits (LoDs) of the mNGS assay were established by evaluating 11 representative microorganisms, comprising two DNA viruses, five hard-to-lyse Gram-positive (G+) bacteria, two Gram-negative (G-) bacteria that are easy to lyse, and two yeast species that are difficult to lyse (Table S1). These strains were introduced into clinically negative plasma at five tenfold serial dilutions, with 20 replicates per dilution. LoD assessments were conducted for each organism using the bioinformatics pipeline, following methods outlined above. The LoD for each organism was determined as the lowest concentration at which detection rate reached or exceeded 95%, with 20 replicates performed for each concentration across all organisms.

### Quantification and statistical analysis

The statistical analysis was carried out using the R software (v4.3.1) (R Core Team, 2021). The Mann–Whitney U test was performed to compare the alpha-diversity indices (ace and chao1 index) between groups. The Wilcoxon rank sum test was performed to compare the beta-diversity assessed by the Bray-Curtis measure. Results were subsequently visualized by principal coordinate analysis (PCoA) plot and principal components analysis (PCA) plot. Differential relative abundance of taxonomic groups at the genus/species level between groups was tested using the Kruskal-Wallis rank sum test (R package “kruskal.test”) (Kruskal and Wallis, 1952). The species and genera with mean relative abundances greater than 1% and penetrance greater than 40% among all samples were compared. In all cases, a two-tailed analysis was performed and considered. Differences were regarded as significant at P < 0.05. Statistical analyses and plots were processed by using SPSS statistical software (IBM SPSS Statistics for Windows, Version 25.0. Armonk, NY, United States) and Origin (Pro) software (Version 2023. OriginLab Corporation, Northampton, MA, USA).
