# Supplementary material for: Optimizing treatment administration strategies using negative mNGS results in corticosteroid-sensitive diffuse parenchymal lung diseases
Source: iScience. 2024 Jun 7;27(7):110218. doi: 10.1016/j.isci.2024.110218 (PMC11237914; doi:10.1016/j.isci.2024.110218)
Supplement: Document S1. Figures S1–S6 and Table S1 [file mmc1.pdf]

iScience, Volume *27*

## Supplemental information

# Optimizing treatment administration strategies using negative mNGS results in corticosteroid-sensitive diffuse parenchymal lung diseases

**Chuwei Jing, Yuchen Ding, Ji Zhou, Qun Zhang, Mingyue Wang, Qiuxiang Ou, Jia Liu, Ting Xv, Chunlai Feng, Dongmei Yuan, Ting Wu, Ting Weng, Xiaoyong Xv, Shanlin Dai, Qian Qian, and Wenkui Sun**

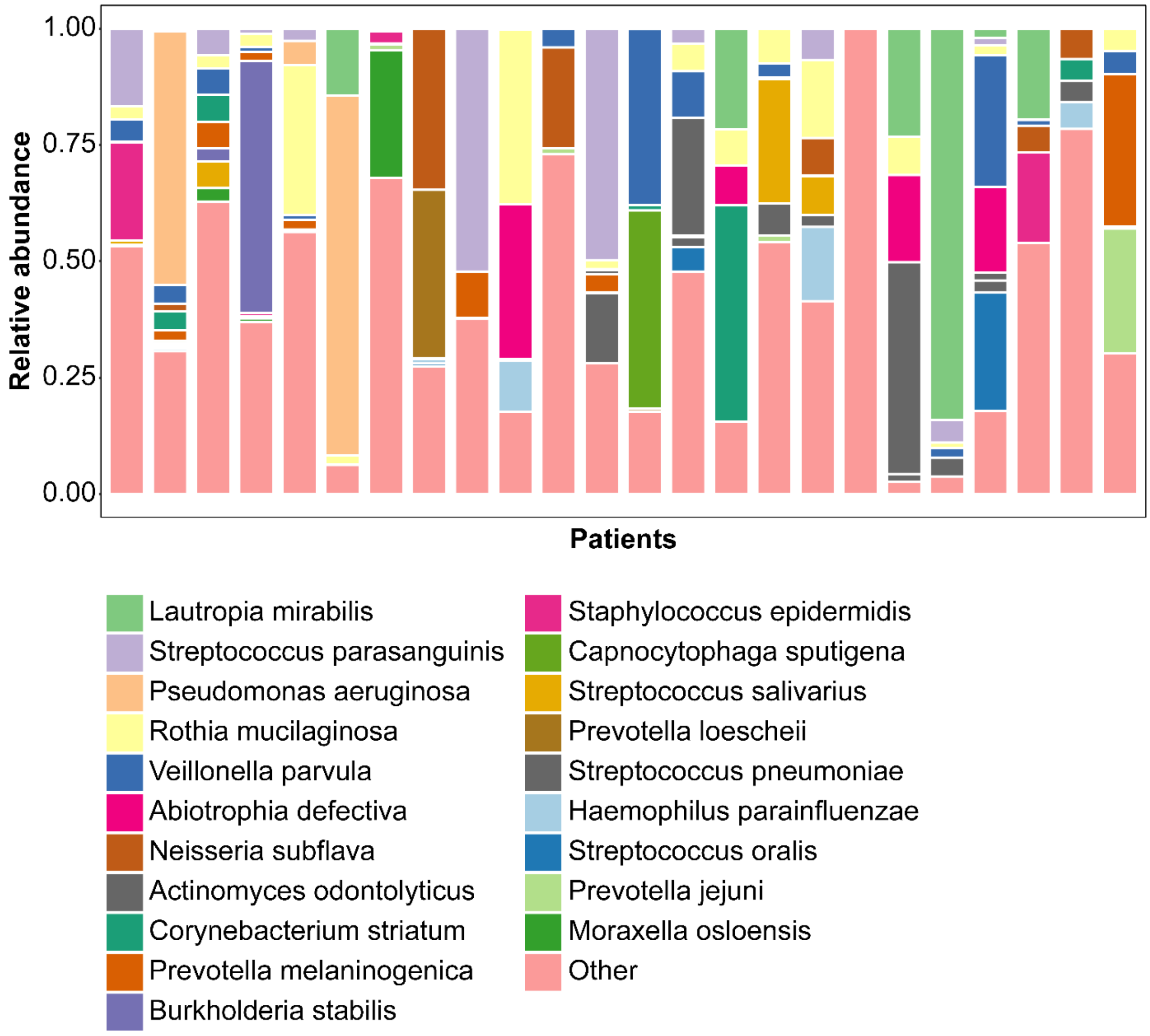


**Figure S1. Lung microbiome of patients with corticosteroid-responsive DPLD**, related to Table 1**.**

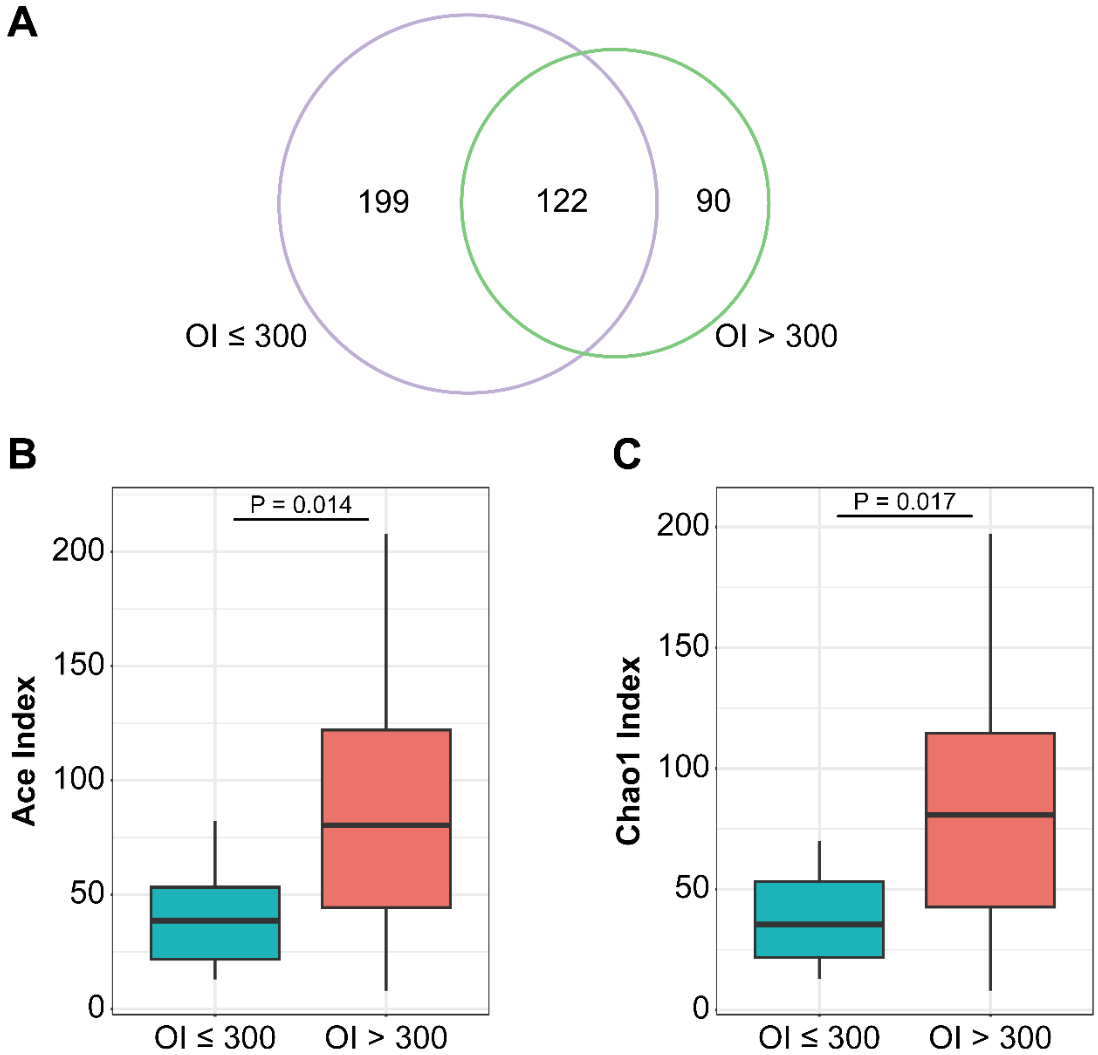


**Figure S2. Detected microbiota and αdiversity analysis between patients with OI above and below 300 (BALF)**, related to Table 1**.** (A) Venn diagram of the detected microbiota (B) Ace Index analysis. (C) Chao1 Index analysis.

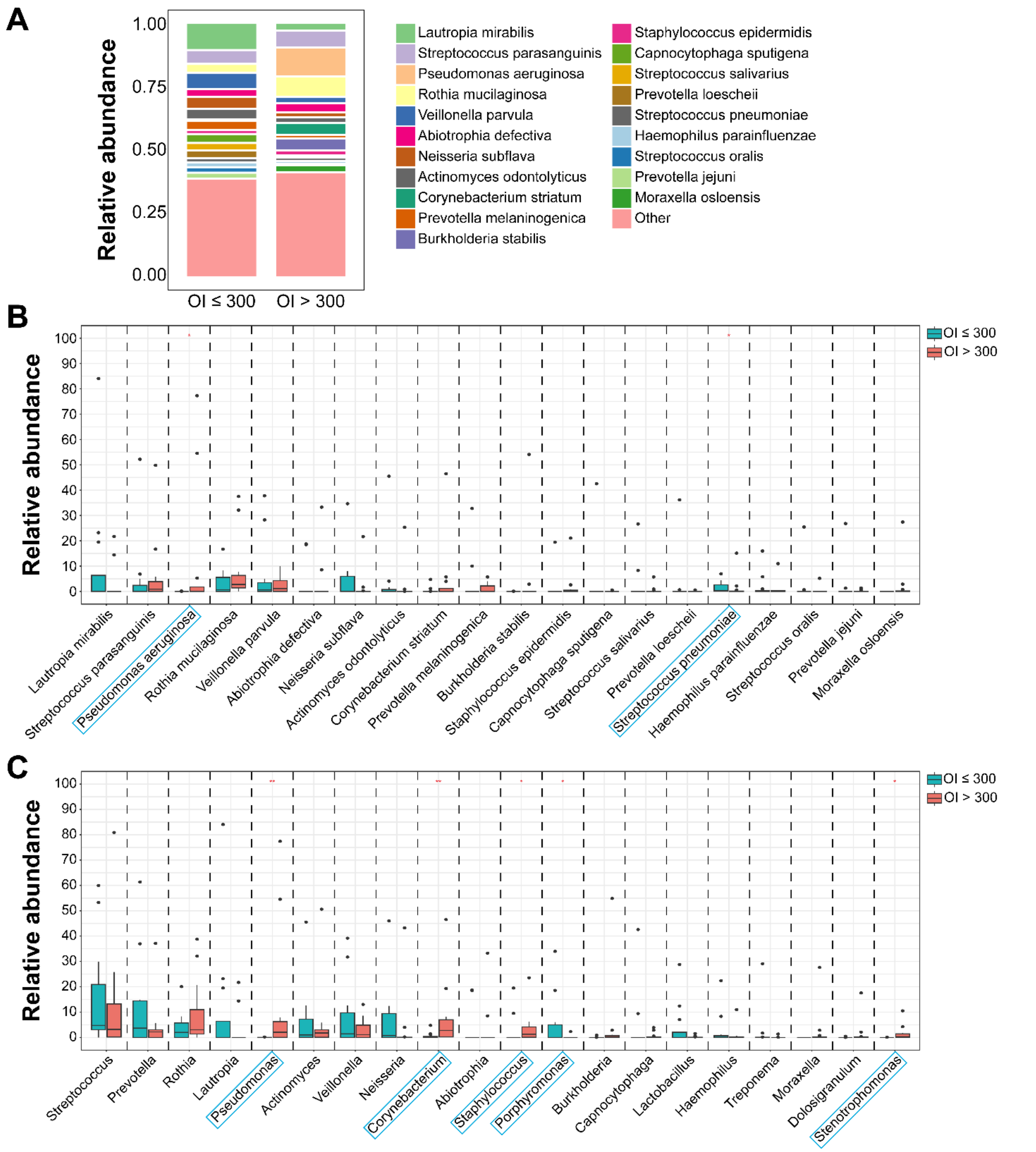


**Figure S3. Comparison of the relative abundance of microorganisms between patients with OI above 300 and below 300**, related to Table 1**.** (A) Distribution of microbiota at the species level. (B) Analysis of significant differences at the genus level. (C) Analysis of significant differences at the species level.

Note: *: P < 0.05; **: P < 0.01; ***: P < 0.001

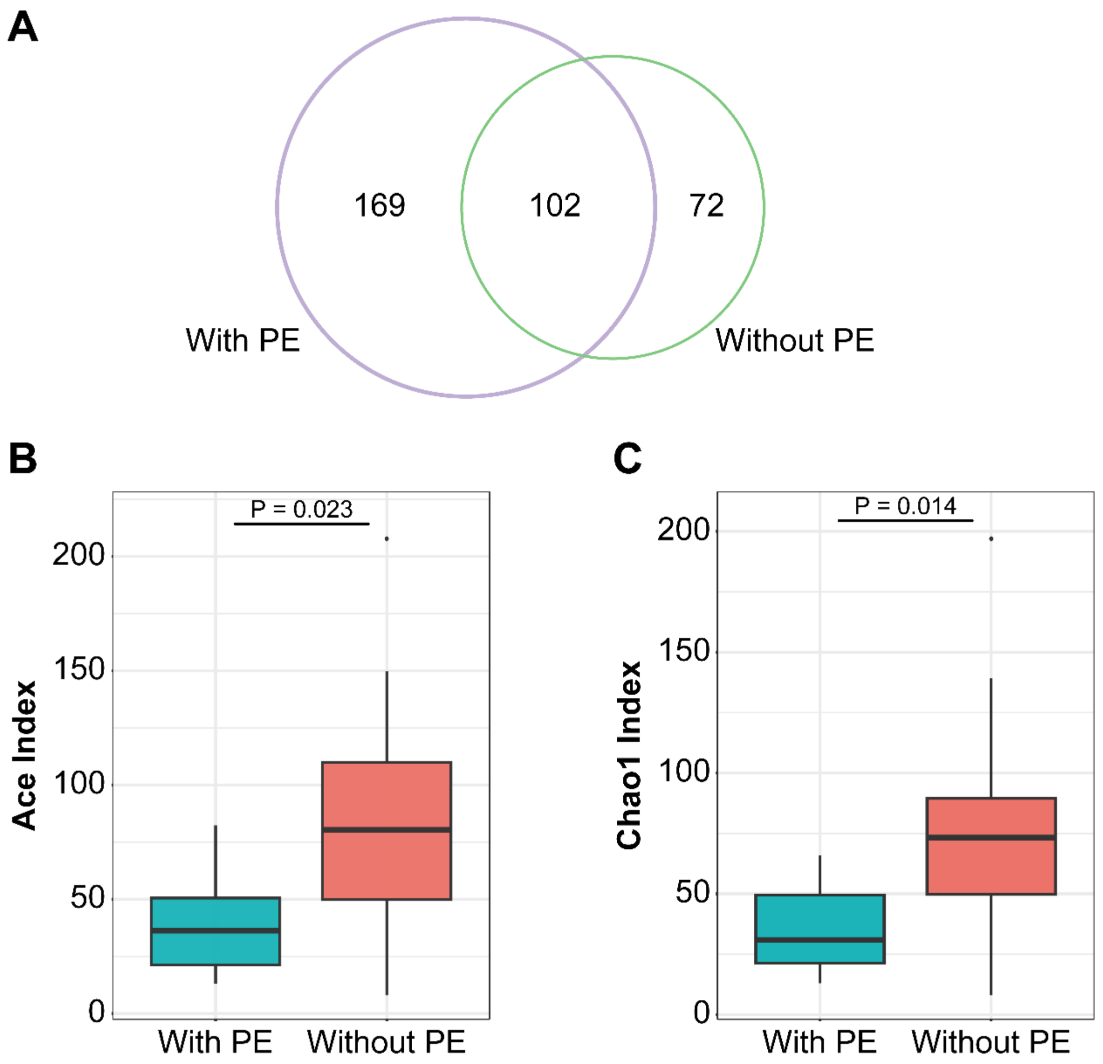


**Figure S4. Detected microbiota and αdiversity analysis between patients with Pleural Effusion and without Pleural Effusion**, related to Table 1**.** (A) Venn diagram of the detected microbiota (B) Ace Index analysis. (C) Chao1 Index analysis.

Note: PE, pleural effusion.

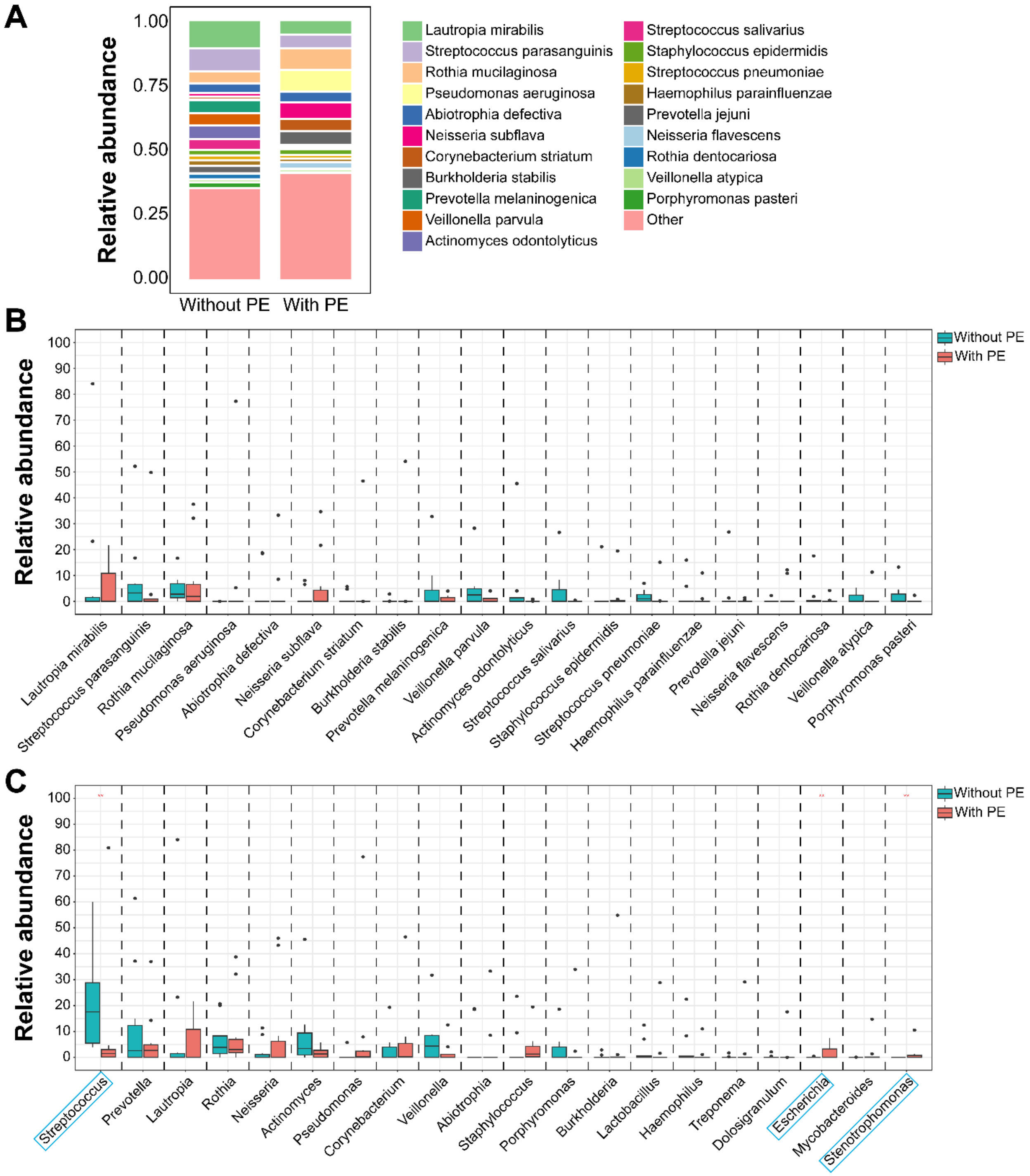


**Figure S5. Comparison of the relative abundance of microorganisms between patients with and without Pleural Effusion**, related to Table 1. (A) Distribution of microbiota at the species level. (B) Analysis of significant differences at the genus level. (C) Analysis of significant differences at the species level.

Note: PE, pleural effusion; *: P < 0.05; **: P < 0.01; ***: P < 0.001

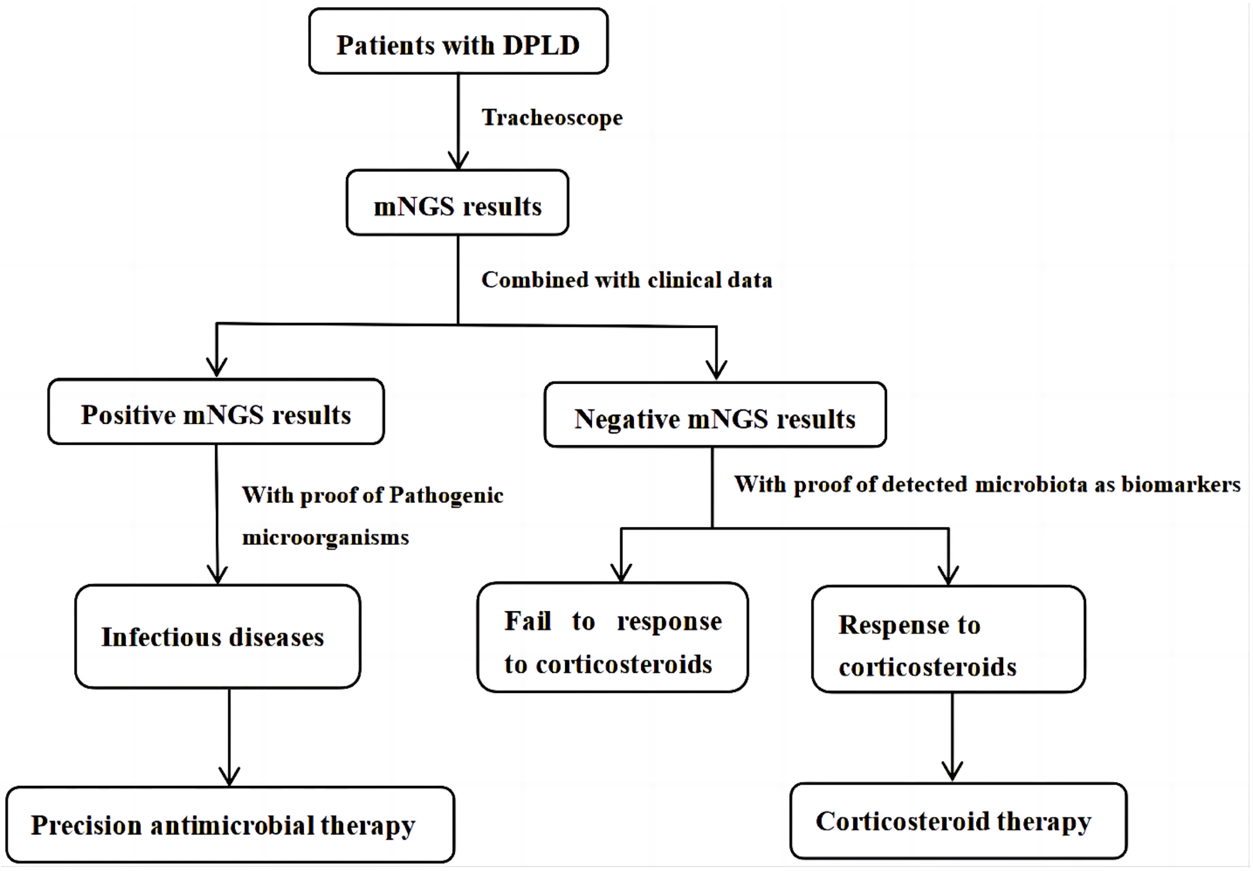


**Figure S6. Flow-chart for suggested clinical management of patients diagnosed with diffuse pulmonary lesions**, related to Figure 3 and Figure 4**.**

**Table S1: Limits of detection of the mNGS for 11 representative pathogens**, related to Figure 1 and STAR Methods**.**

| Subgroup | Type | Species | Limit of detection (copies/mL) | Genome size (Mb) |
|---|---|---|---|---|
| Bacteria | G+ | *Bacillus subtilis* | 270 | 4.22 |
| | G+ | *Staphylococcus aureus* | 40 | 2.82 |
| | G+ | *Listeria monocytogenes* | 37 | 2.94 |
| | G+ | *Lactobacillus fermentum* | 570 | 1.93 |
| | G+ | *Enterococcus faecalis* | 385 | 2.87 |
| | G− | *Salmonella enterica* | 230 | 4.86 |
| | G− | *Pseudomonas aeruginosa* | 160 | 6.26 |
| Fungi | – | *Saccharomyces cerevisiae* | 150 | 11.83 |
| | – | *Cryptococcus neoformans* | 10 | 18.56 |
| Virus | – | *Cytomegalovirus* | 1800 | 0.23 |
| | – | Adenovirus type 35 | 16,000 | 0.035 |
